# Effective Treatment of Macrophage Activation Syndrome Linked to Systemic Lupus Erythematosus Using Interleukin-1 Inhibitor

**DOI:** 10.7759/cureus.82379

**Published:** 2025-04-16

**Authors:** Nur Barlas, Ikwinder P Kaur, Cristine Kuzhuppilly Arcilla, Gurjit S Kaeley, Myint Thway

**Affiliations:** 1 Internal Medicine, Florida State University College of Medicine, Cape Coral, USA; 2 Rheumatology, University of Florida College of Medicine – Jacksonville, Jacksonville, USA; 3 Internal Medicine/Rheumatology, University of Florida College of Medicine – Jacksonville, Jacksonville, USA

**Keywords:** anakinra, hemophagocytic lymphohistiocytosis, interleukin-1 inhibitor, macrophage activation syndrome, systemic lupus erythematosus

## Abstract

Macrophage activation syndrome (MAS) is a severe hyperinflammatory condition often associated with autoimmune diseases, particularly systemic lupus erythematosus (SLE). It presents significant diagnostic challenges due to overlapping clinical features with SLE flares. This case report presents the diagnostic complexities of MAS in a 19-year-old female with SLE and lupus nephritis. She initially presented with persistent fever, cytopenias, hyperferritinemia, hypertriglyceridemia, and elevated inflammatory markers. Despite comprehensive evaluation, including a bone marrow biopsy that did not reveal hemophagocytosis, MAS was diagnosed based on clinical and laboratory findings. Initial treatment with high-dose intravenous steroids led to temporary improvement, but symptoms recurred upon tapering. Subsequently, the patient was treated with Anakinra, an interleukin-1 receptor antagonist, resulting in rapid clinical recovery and normalization of laboratory values. She remained on Anakinra for six months without experiencing a recurrence of MAS. This case underscores the diagnostic challenges of identifying MAS in patients with SLE and suggests that Anakinra may be an effective treatment option. It highlights the need for further research to refine management strategies for this potentially life-threatening condition.

## Introduction

Macrophage activation syndrome (MAS) is a rare but life-threatening hyperinflammatory condition that occurs as a secondary form of hemophagocytic lymphohistiocytosis (HLH). It is characterized by excessive immune activation, leading to uncontrolled cytokine release and multiorgan dysfunction [1]. MAS most commonly complicates systemic juvenile idiopathic arthritis (SJIA) but can also occur in other autoimmune diseases, including systemic lupus erythematosus (SLE). The estimated prevalence of MAS in SLE patients ranges from 0.9% to 9%, with a reported mortality rate between 8% and 22% due to delayed recognition and treatment [2,3].

The pathophysiology of MAS involves dysregulated macrophage and cytotoxic T-cell activation, resulting in a “cytokine storm” driven by interleukin (IL)-1β, IL-6, tumor necrosis factor-alpha (TNF-α), and interferon-gamma (IFN-γ), which contribute to severe systemic inflammation, persistent fever, cytopenias, hyperferritinemia, and multiorgan dysfunction [1,4,5]. Elevated IL-18 and CXCL9 levels further distinguish MAS from other inflammatory conditions and may serve as useful biomarkers [6].

Diagnosing MAS in SLE patients is particularly challenging due to its significant clinical overlap with lupus flares. Both conditions can present with fever, cytopenias, liver dysfunction, and coagulopathy, making early differentiation crucial for appropriate management [1]. Extreme hyperferritinemia, hypofibrinogenemia, and highly elevated soluble IL-2 receptor levels are key distinguishing features of MAS in SLE patients [4,6]. However, the absence of hemophagocytosis on bone marrow biopsy does not exclude MAS, emphasizing the importance of a comprehensive clinical and laboratory-based diagnosis [6,7].

SLE is a chronic autoimmune disorder characterized by immune dysregulation, autoantibody production, and systemic inflammation affecting multiple organ systems [8]. One of the key immunopathogenic mechanisms in SLE involves loss of immune tolerance, leading to persistent B- and T-cell activation and an excessive type I interferon response. This results in immune complex deposition, complement activation, and sustained inflammation, contributing to widespread tissue damage [5,8]. MAS in SLE is often triggered by infections, disease flares, or immunosuppressive therapy, making diagnosis even more challenging [9].

This case report describes a 19-year-old female with SLE complicated by MAS, highlighting the diagnostic challenges and therapeutic considerations. The patient exhibited moderate SLE activity without hepatosplenomegaly or neurological involvement, yet developed MAS, reinforcing the need for high clinical suspicion in febrile SLE patients with worsening cytopenias [10]. Notably, the patient responded rapidly to Anakinra (an IL-1 receptor antagonist), achieving sustained remission, demonstrating the potential role of IL-1 blockade as an early therapeutic option in MAS-SLE [6,11].

This case contributes to the growing evidence supporting early intervention in MAS and underscores the need for further research to refine MAS diagnostic criteria and optimize treatment strategies in SLE patients [12].

## Case presentation

Clinical history

A 19-year-old female with a known history of SLE and recently diagnosed Class II/III lupus nephritis presented with altered mental status, persistent high-grade fevers, and profound fatigue. Neuropsychiatric symptoms included auditory hallucinations, paranoid ideation, erratic behavior, and insomnia for two days. She denied visual hallucinations. The exact duration of the fever was unclear. She had been diagnosed with SLE three years earlier and was initially managed with hydroxychloroquine (200 mg twice daily). Three weeks before this admission, she underwent a kidney biopsy that confirmed Class II/III lupus nephritis. Treatment was intensified with mycophenolate mofetil (500 mg twice daily), hydroxychloroquine (200 mg twice daily), and prednisone (60 mg daily).

Physical examination

Vital signs on admission were as follows: blood pressure of 135/73 mmHg, heart rate of 115 beats per minute, respiratory rate of 18 breaths per minute, oral temperature of 36.9°C (98.5°F), and oxygen saturation of 99% on room air. Her last menstrual period was two weeks prior. She weighed 70 kg. She appeared well-developed and well-nourished, alert but intermittently cooperative and non-verbal, and in no acute distress. Her cardiovascular, respiratory, and musculoskeletal exams were unremarkable. Neurologically, she was oriented with intact memory and language; her attention was mildly impaired. Cranial nerves II-XII were intact, motor strength was 5/5, and reflexes were 2+ and symmetric. No sensory deficits or cerebellar abnormalities were noted. No meningeal signs were present.

Differential diagnosis

The differential diagnosis included lupus cerebritis, central nervous system (CNS) infection, MAS, steroid-induced psychosis, autoimmune encephalitis, and acute brief psychotic disorder.

Diagnostic evaluation

**Figure 1:**
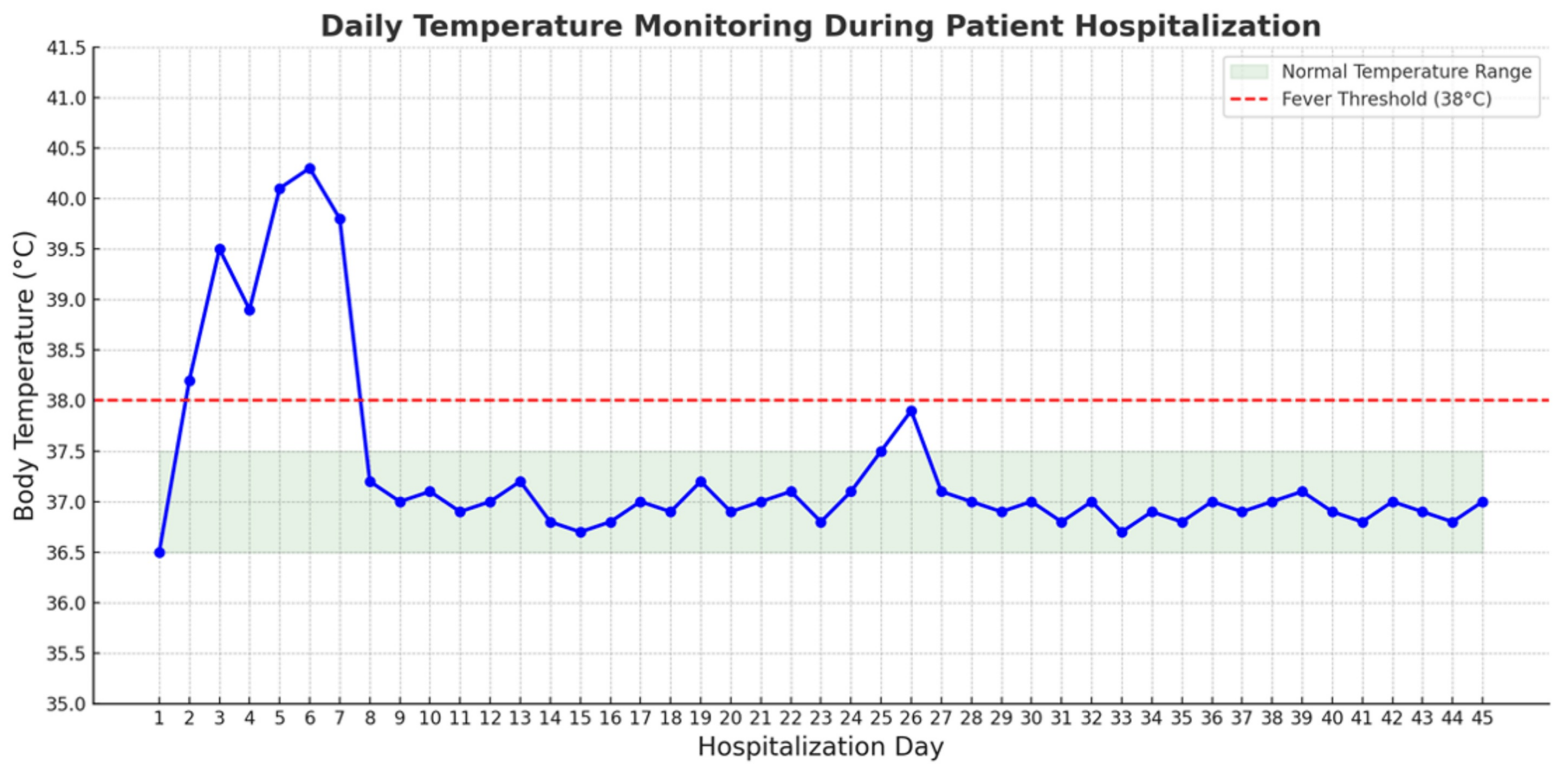
Fever trend of the patient during hospitalization.

Neurological and infectious workup included a 20-minute electroencephalography, which showed no abnormalities. The encephalitis panel was negative. Non-contrast CT of the head revealed no hemorrhage, mass, or infarct. MRI of the brain showed no evidence of demyelination or structural lesions. Cerebrospinal fluid studies showed normal glucose, protein, cell count, and opening pressure. Gram stain and viral PCRs were negative (including herpes simplex virus, Epstein-Barr virus, cytomegalovirus). A toxicology screen, human immunodeficiency virus, and syphilis testing were negative. Blood cultures and a comprehensive infectious panel were also negative. Despite broad-spectrum antibiotics and corticosteroids, she remained febrile (peaking at 39.5°C on day three), suggesting a cytokine-driven hyperinflammatory process rather than infection. Persistent high-grade fevers (peaking at 39.5°C) were noted despite broad-spectrum antimicrobial therapy and corticosteroids (Figure 1).

Laboratory findings

Severe normocytic anemia with a hemoglobin level of 7.5 g/dL was noted. The reticulocyte index was 0.4%, indicating inadequate bone marrow compensation rather than hemolysis. Haptoglobin was normal, and DAT was negative, suggesting non-hemolytic anemia. The white blood cell count (WBC) was 1.19 × 10³/μL, suggesting leukopenia. Anti-dsDNA was elevated at >300 IU/mL (reference range: 0-9 IU/mL). Complement C3 was low at 55 mg/dL (reference range: 90-180 mg/dL). Moreover, proteinuria was noted (1,131 mg on urinalysis).

An expert hematologist reviewed the bone marrow biopsy, and they were informed of our suspicion of HLH/MAS. Bone marrow biopsy revealed hypocellular marrow (50-60%) with preserved trilineage hematopoiesis and no evidence of hemophagocytosis, infiltrative disease, or blasts. Notably, hemophagocytosis is absent in up to 40% of MAS cases. These findings suggested that the anemia was primarily driven by the underlying inflammatory state of MAS rather than a nutritional deficiency or hemolysis.

Additional findings

Neutropenia (WBC was 1.19 × 10³/μL on admission), low complement C3 (55 mg/dL; reference range: 90-180 mg/dL), and elevated anti-dsDNA (>300 IU/mL; reference range: 0-9 IU/mL) confirmed active SLE flare. Urinanalysis showed urine protein at 1,131 mg. Given the persistent fevers, cytopenias, and elevated inflammatory markers, MAS was suspected. The following additional laboratory markers supported this diagnosis: ferritin: 3,520 ng/mL (reference range: 15-150 ng/mL), consistent with anemia of inflammation rather than iron deficiency; triglycerides: 366 mg/dL (reference range: <150 mg/dL); fibrinogen: 183 mg/dL (reference range: 186-461 mg/dL); aspartate aminotransferase: 108 IU/L (reference range: 10-40 IU/L); and soluble IL-2 receptor (sCD25): 757 U/mL (reference range: 223-710 U/mL).

These findings fulfilled several HLH-2004 and European League Against Rheumatism (EULAR)/American College of Rheumatology (ACR) criteria for MAS, supporting the diagnosis despite a negative bone marrow biopsy. Lupus flares and sepsis may mimic MAS, but the constellation of persistent fevers, hyperferritinemia, hypertriglyceridemia, and cytopenias favored MAS.

Treatment and clinical course

The patient was initially managed with melatonin and olanzapine 5 mg twice daily for neuropsychiatric symptoms. Due to poor oral intake, she was transitioned to intramuscular olanzapine. Broad-spectrum antibiotics (meropenem) and neutropenic precautions were also initiated.

On hospital day two, high-dose intravenous corticosteroids (1 g methylprednisolone daily for three consecutive days) were initiated to suppress hyperinflammation and macrophage overactivation. The patient showed initial clinical improvement, including reduced agitation and stabilization of inflammatory markers. However, fevers recurred on hospital day five after completing the pulse steroid course, as the steroid taper began. This indicated steroid-refractory MAS and prompted reassessment of the treatment plan.

Given the pathophysiologic role of IL-1β in MAS, Anakinra (100 mg subcutaneously daily) was initiated as targeted cytokine therapy. The patient exhibited rapid clinical improvement within 48 hours. Her fevers resolved, inflammatory markers began to normalize, and mental status significantly improved. She continued on Anakinra for six months with no recurrence of MAS features.

This case supports the growing recognition of Anakinra as an effective cytokine-targeted therapy for steroid-refractory MAS in patients with SLE. In SLE patients presenting with persistent fevers, cytopenias, and systemic inflammation unresponsive to standard therapy, MAS should be strongly considered, even in the absence of hemophagocytosis [3]. This case highlights the utility of early Anakinra initiation and the need for further studies to refine diagnostic biomarkers that differentiate MAS and SLE flares.

**Figure 2:**
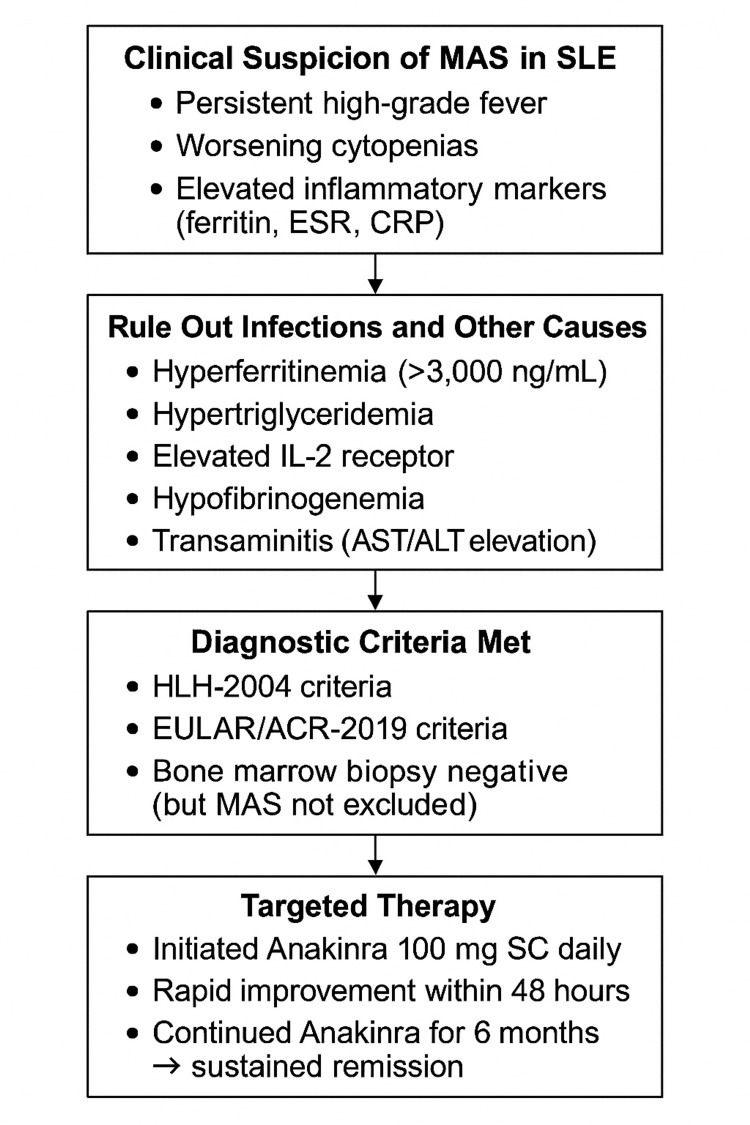
Diagnostic and therapeutic approach for MAS in SLE. MAS: macrophage activation syndrome; SLE: systemic lupus erythematosus; ESR: erythrocyte sedimentation rate; CRP: C-reactive protein; AST: aspartate aminotransferase; ALT: alanine aminotransferase; HLH: hemophagocytic lymphohistiocytosis; EULAR/ACR: European League Against Rheumatism/American College of Rheumatology

Given the patient’s persistent fever, cytopenias, and elevated inflammatory markers, MAS was suspected. Additional laboratory markers confirmed the diagnosis, leading to high-dose corticosteroid treatment. Figure 2 outlines the diagnostic and therapeutic approach used in this case.

## Discussion

MAS is a severe and potentially life-threatening complication associated with various rheumatologic diseases, particularly SJIA [13]. MAS is a form of HLH seen in rheumatic disorders, caused by dysregulated immune activation and excessive cytokine production (IL-1β, TNF, IFN-γ), leading to hyperinflammation and tissue damage.

Risk assessment tools such as the HLH score (Table 1) and the EULAR/ACR 2016 MAS Classification Criteria aid in diagnosis (Table 2) [1]. The EULAR/ACR developed the 2016 Classification Criteria for MAS, focusing primarily on laboratory features [1].

**Table 1:** The 2004 HLH classification criteria for MAS. HLH: hemophagocytic lymphohistiocytosis; MAS: macrophage activation syndrome; NK: natural killer; IL-2: interleukin-2

Diagnostic criteria (must fulfill five of the eight criteria below)
Fever
Splenomegaly
Cytopenia (affecting two of three lineages)
Hemoglobin <90 g/dL
Platelets <100 x 10⁹/L
Neutrophils <0.1 x 10⁹/L
Hypertriglyceridemia or hypofibrinogenemia
Fasting triglycerides >285 mg/dL
Hemophagocytosis in bone marrow, spleen, or lymph nodes
Low or absent NK cell activity
Soluble IL-2 receptor >2,400 U/mL

**Table 2:** The 2016 EULAR/ACR classification criteria for MAS. EULAR/ACR: European League Against Rheumatism/American College of Rheumatology; MAS: macrophage activation syndrome; AST: aspartate aminotransferase

Diagnostic criteria
Ferritin >684 ng/mL
And any two of the following:
Platelet count <181 × 10^9^/L
AST >48 U/L
Triglycerides >156 mg/dL
Fibrinogen <360 mg/dL

In our case, the patient met the clinical and laboratory criteria for MAS despite a negative bone marrow biopsy. Previous reports emphasize that clinical and biochemical markers must guide diagnosis rather than relying solely on histopathologic findings, as hemophagocytosis is not always present in MAS [10].

This case adds to the growing evidence supporting the efficacy of moderate-dose Anakinra (100 mg daily) in MAS secondary to SLE. Unlike prior studies that documented high-dose Anakinra (e.g., 400 mg daily or 8 mg/kg/day) for severe MAS [6], our case demonstrates that a lower dose achieved sustained remission, reinforcing the importance of individualized treatment approaches. This case is one of the few reports of early moderate-dose Anakinra (100 mg daily) in non-refractory MAS-SLE. Prior studies primarily explored high-dose Anakinra (400 mg daily or 8 mg/kg/day) for severe or steroid-refractory MAS, making this case significant in showing that early IL-1 blockade at a lower dose may prevent escalation to high-dose immunosuppression. This insight challenges current treatment assumptions and suggests that future guidelines may consider moderate-dose Anakinra as a viable first-line strategy in select MAS-SLE cases. Meneghel et al. reported successful use of high-dose intravenous Anakinra in a case of fulminant myocarditis-associated MAS, supporting its role in life-threatening presentations [14]. Gullickson et al. described continuous Anakinra infusion as a novel therapeutic approach for severe, rapid disease control in refractory MAS [15].

Unlike past reports requiring high-dose Anakinra for refractory cases, this case achieved remission with 100 mg daily, without dose escalation [16]. This highlights its potential for rapid cytokine suppression in critical settings. Halyabar et al. reported that Anakinra at 8 mg/kg/day rapidly normalized MAS biomarkers [11], while Eloseily et al. emphasized that high-dose Anakinra was required for disease severity, and early intervention may play a crucial role in determining optimal Anakinra dosing [6]. Unlike prior cases requiring high-dose Anakinra for severe multi-organ involvement, this patient avoided life-threatening complications such as acute respiratory distress syndrome or liver failure. This raises a key question: could earlier, lower-dose intervention prevent MAS progression? While high-dose Anakinra is well-documented for steroid-refractory MAS, data on moderate doses in mild-to-moderate MAS-SLE are limited. This case suggests that early IL-1 blockade at a lower dose may effectively treat MAS and prevent escalation. Future studies should explore its role in MAS-SLE management before high-dose therapy becomes necessary. Our case contrasts with these by achieving remission with moderate-dose Anakinra (100 mg daily), suggesting that earlier IL-1 blockade at a lower dose may be sufficient to halt disease progression while minimizing immunosuppressive risks.

Compared to cases where tocilizumab or cyclosporine were used, this case highlights Anakinra’s rapid efficacy in steroid-refractory MAS, with a faster onset and a favorable safety profile [17]. Tocilizumab, an IL-6 inhibitor, has been effective in MAS, but its use remains controversial due to its limited impact on hyperferritinemia [18]. Cyclosporine, a calcineurin inhibitor, has been widely used in MAS but carries higher toxicity risks, particularly nephrotoxicity. By demonstrating effective disease control with moderate-dose Anakinra, this case suggests that IL-1 blockade may be a viable first-line option in MAS-SLE, particularly in non-refractory cases.

IL-1β plays a key role in MAS pathogenesis by promoting macrophage activation and hyperinflammation. The excessive cytokine production seen in MAS results in a cytokine storm, leading to fever, cytopenias, hyperferritinemia, and multi-organ dysfunction.

Anakinra, an IL-1 receptor antagonist, blocks IL-1β early, stopping further cytokine activation [6]. Unlike IL-6 inhibitors, which primarily block downstream inflammation, IL-1 blockade targets the initial inflammatory cascade, making early intervention crucial.

IL-1β drives hyperferritinemia, a hallmark of MAS, leading to prolonged macrophage activation and tissue damage [6]. Early Anakinra administration may prevent ferritin-driven immune dysregulation, reducing the risk of multi-organ failure. Anakinra has been shown to reduce macrophage activation, preventing the uncontrolled immune response that characterizes MAS [11].

Our case supports early IL-1 blockade, even in moderate MAS-SLE cases, challenging the assumption that high-dose Anakinra is always required for remission.

This case has several limitations that should be acknowledged. Hemophagocytosis is often considered a hallmark of MAS, but its absence does not exclude the diagnosis [1]. Previous reports indicate that up to 40% of MAS cases lack bone marrow hemophagocytosis at presentation [10]. Clinicians should prioritize biochemical markers such as ferritin, triglycerides, and fibrinogen over bone marrow findings when diagnosing MAS.

MAS is a relapsing condition, and longer follow-up is necessary to assess remission durability and relapse risk. While based on pediatric cases, Dey et al. suggested that recurrence is not uncommon in MAS-SLE [19]. Future research should include longer follow-up periods and genetic testing to predict relapse risk and tailor long-term management strategies.

MAS presents with heterogeneous clinical manifestations, and while this patient responded well to moderate-dose Anakinra, other cases may require higher dosing or combination therapy. Future studies should define optimal Anakinra dosing strategies based on disease severity, inflammatory markers, and timing of intervention.

## Conclusions

This case supports the use of early, moderate-dose Anakinra in MAS-SLE, showing it may achieve remission while minimizing immunosuppression. Future studies should explore optimal dosing strategies through multicenter research.
